# Immediate and Delayed Effects of Cupping Therapy on Reducing Neuromuscular Fatigue

**DOI:** 10.3389/fbioe.2021.678153

**Published:** 2021-07-01

**Authors:** Xiao Hou, Xiaoling Wang, Lisa Griffin, Fuyuan Liao, Joseph Peters, Yih-Kuen Jan

**Affiliations:** ^1^Department of Sports Science and Physical Education, Tsinghua University, Beijing, China; ^2^Department of Kinesiology and Community Health, University of Illinois at Urbana-Champaign, Champaign, IL, United States; ^3^College of Rehabilitation Medicine, Fujian University of Traditional Chinese Medicine, Fuzhou, China; ^4^Department of Kinesiology and Health Education, University of Texas at Austin, Austin, TX, United States; ^5^Department of Biomedical Engineering, Xi’an Technological University, Xi’an, China

**Keywords:** cupping therapy, delayed effect, electromyography, immediate effect, muscle fatigue

## Abstract

Cupping therapy has been popular in elite athletes in recent years. However, the effect of cupping therapy on reducing muscle fatigue has not been investigated. The purpose of this study was to investigate the immediate and delayed effects of cupping therapy on reducing biceps brachii fatigue during biceps curls. Twelve healthy untrained participants were recruited for this repeated-measures study. Cupping therapy (–300 mmHg pressure for 5 min) and sham control (no negative pressure for 5 min) were applied after biceps fatigue induced by performing repeated biceps curls at 75% of the 10 repetitions of maximum of the non-dominant hand. Surface electromyography (EMG) with spectral analyses [mean frequency (MNF), median frequency (MDF), and spectral moments ratio (SMR)] were used to assess muscle fatigue during the fatigue task. EMG signals during the first 10 repetitions and the last 10 repetitions of biceps curls were used to assess neuromuscular fatigue. There were significant decreases in MNF and MDF and a significant increase in SMR immediately and 24 h after the sham control (no intervention). When comparing the MNF, MDF, and SMR after cupping therapy to the sham control, there was no significant immediate effect on reducing muscle fatigue. However, there was a significant delayed effect on improving recovery following fatigue for the cupping therapy compared to the sham control (MNF changes: sham 0.87 ± 0.02 vs. cupping 0.91 ± 0.02, *p <* 0.05; MDF changes sham: 0.85 ± 0.03 vs. cupping: 0.91 ± 0.02, *p <* 0.05; SMR changes: sham 1.89 ± 0.15 vs. cupping 1.58 ± 0.13, *p <* 0.05). The findings of this study demonstrate that there is a time effect of cupping therapy for reducing muscle fatigue. Cupping therapy is effective on reducing biceps brachii muscle fatigue after 24 h.

## Introduction

Muscle fatigue is defined as a transient reduction in the capacity to perform physical action (Enoka and Duchateau, 2008). The potential mechanisms of muscle fatigue include the accumulation of metabolites within the muscle and the generation of insufficient motor commands from the motor cortex during exercise (Enoka and Duchateau, 2008). Severe muscle fatigue can significantly affect sports performance and joint stability, as well as increase risk of sports injury (Goodall et al., 2015; Van Hooren and Peake, 2018). Therefore, it is important to provide effective interventions for improving recovery of muscle fatigue after exercise.

Various interventions (e.g., stretching, cooling, and massage) have been proposed to reduce muscle fatigue after exercise (Wiltshire et al., 2010; De Pauw et al., 2011). Post-exercise stretching can reduce muscle soreness (Herbert and de Noronha, 2007), but it can cause a decrease in muscle strength (Power et al., 2004; Behm and Chaouachi, 2011). Cooling demonstrates promise on reducing muscle soreness after exercise, but it may delay the healing process (Hohenauer et al., 2015). Massage, a widely used intervention for reducing muscle fatigue in sports competitions, can alleviate muscle soreness after intensive exercise, but it has no recovery effect on muscle strength (Hunter et al., 2006; Bender et al., 2019). Although these three popular interventions may ameliorate muscle fatigue, they may cause adverse effects on muscle recovery and performance. Therefore, an intervention capable of reducing muscle fatigue without causing significant adverse effects should be identified for reducing risk of sports injury associated with muscle fatigue.

Cupping therapy has been popular in elite athletes (e.g., Michael Phelps, Alex Naddour, and Pavel Sankovich) in recent years (RIO, 2016; Chen et al., 2018; Chiu et al., 2020). Compared with commonly used interventions (e.g., stretching, cooling, and massage), cupping therapy demonstrates promise for reducing muscle fatigue without causing adverse effects on the muscle (Lowe, 2017; Hou et al., 2020; Wang et al., 2020; Jan et al., 2021). Cupping therapy can improve local blood flow (Hou et al., 2020; Wang et al., 2020), alleviate muscle pain (Kim et al., 2012; Lauche et al., 2012; Al-Bedah et al., 2015), and reduce muscle stiffness (Jan et al., 2021). All of these therapeutic effects associated with cupping therapy may benefit recovery of exercise-induced muscle fatigue. Therefore, cupping therapy may be a better intervention for improving recovery from muscle fatigue than commonly used interventions. However, the effect of cupping therapy on reducing muscle fatigue has not been investigated.

Exercise-induced muscle fatigue is a continuous process of gradually decreasing performance of a muscle (Cifrek et al., 2009). It is not currently feasible to continuously measure fatigue-related biochemical biomarkers [e.g., plasma creatine kinase (CK) and lactate] of an active muscle during exercise. Surface electromyography (EMG), a local, non-invasive and real-time recording, can continuously measure myoelectric activity of a muscle during the process of muscle fatigue (Cifrek et al., 2009; Zhang et al., 2018). Continuous EMG signals may be more related to the real-time process of muscle fatigue compared to biomarkers drawn from one time point after exercise. Several studies have demonstrated that EMG signals, including median frequency (MDF) and mean frequency (MNF), can be used to assess muscle fatigue (De Luca, 1984).

Interventions used to improve recovery from muscle fatigue invoke time-dependent responses that have both immediate and delayed effects (Hilbert et al., 2003; Zainuddin et al., 2005). Hilbert et al. found that the recovery effect of massage on muscle soreness did not appear until 48 h after exercise (Hilbert et al., 2003). Similarly, massage caused a significant decrease in CK 4 days after exercise, but there was no significant difference in CK prior to that after exercise (Zainuddin et al., 2005). Muscle fatigue induced by intensive exercise may cause an inflammatory response (Theofilidis et al., 2018). Massage may promote the release of anti-inflammatory cytokines (e.g., neutrophil) from blood vessel walls through the elevation of mechanical shear stress (Smith et al., 1994). However, such anti-inflammatory response induced by massage may take up to 5 days to modulate the inflammatory response after exercise. Cupping therapy, like massage, is a physical intervention that promotes a healing effect by applying mechanical stress (i.e., negative pressure) to the soft tissue (Tham et al., 2006; Wang et al., 2020; Jan et al., 2021). The negative pressure of cupping therapy can increase the shear stress within blood vessels to generate endogenous anti-inflammatory cytokines in response to the inflammation after exercise. Therefore, we hypothesized that there is a time effect in cupping therapy on improving muscle recovery following fatigue.

The objective of this study was to investigate the immediate and delayed effect of cupping therapy on muscle fatigue EMG measures. Specifically, we hypothesized that cupping therapy has a delayed effect, but not an immediate effect on reducing exercise-induced muscle fatigue.

## Materials and Methods

### Participants

Twelve healthy, untrained individuals (6 males and 6 females) aged 18–40 years of age were recruited. Exclusion criteria consisted of diagnosed ischemic heart diseases (diagnosed coronary insufficiency, arrhythmia, and heart failure), diagnosed diabetes mellitus, vascular diseases, neuromuscular impairments, and hypertension [systolic blood pressure (SBP) ≥ 140 mmHg or diastolic blood pressure (DBP) ≥ 90 mmHg]. Participants who had a non-blanchable response of the red skin area over the biceps (non-dominant side), open wound, scar, or tattoo over the tested area were also excluded. Participants who were athletes or had previously received cupping therapy were excluded.

Participants were recruited from the University of Illinois at Champaign-Urbana through flyers and word-of-mouth. All participants signed an informed consent approved by the University of Illinois at Urbana-Champaign Institutional Review Board (#20423). The sample size was determined to be 12 based on the power analysis with an assumption of a very large effect size (1.2), alpha level at 0.05, and power at 0.95 for the repeated measures study. The selection of a very large effect size was based on relevant studies (He et al., 2020; Wang et al., 2020) showing a very large effect of cupping therapy on increasing blood flow. A significant increase in blood flow after cupping therapy may effectively improve recovery from muscle fatigue.

### Cupping Therapy Dose

A 45 mm (inner diameter) cup with a rounded rim was used to apply cupping therapy (He et al., 2020). The cup had a rim width of 4 mm for a total outer diameter of 53 mm. The rounded rim of the cup was used to avoid causing sharp pain to the individual during the cupping therapy (Kravetz, 2004). In consideration of biceps brachii size and the commonly used sizes of inner diameters between 38 and 51 mm (Jan et al., 2021), a 45-mm cup was chosen for both male and female participants in this study.

In this study, we chose –300 mmHg for cupping therapy because a negative pressure between –225 and –375 mmHg is commonly applied to treat musculoskeletal impairments (Al-Bedah et al., 2016). We chose 5 min for the duration of cupping therapy because previous studies have used durations ranging from 5 to 20 min for different impairments (Sanders et al., 1995; Tham et al., 2006; Dayer-Berenson, 2010; Roostayi et al., 2016). Our previous studies demonstrated that cupping therapy at –300 mmHg for 5 min could significantly increase skin blood flow of the upper arm (Hou et al., 2020). Thus, we used cupping therapy at –300 mmHg for 5 min to reduce muscle fatigue in this study. An electric device was used to apply negative pressure (Powerpress, Chatsworth, CA). Sham cupping therapy (i.e., no negative pressure applied) was used as the control (Wang et al., 2017); double-sided tape was used on the rim of the cup and the skin under the rim for the sham cupping therapy.

### Study Design

**FIGURE 1:**
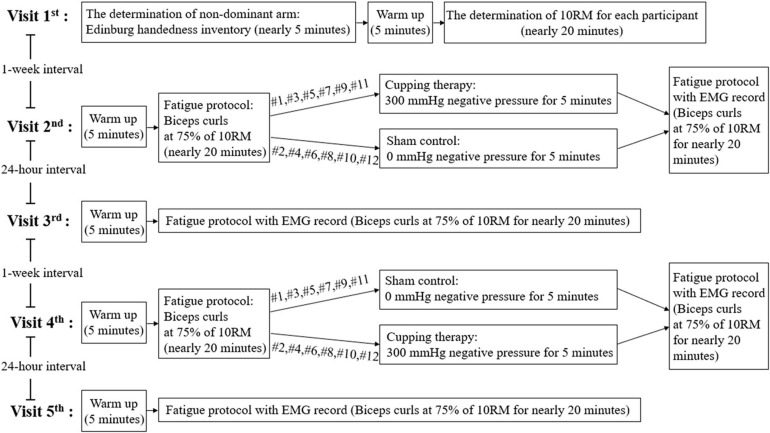
The study design and experimental protocol.

A repeated-measures design consisting of 5 visits was used in this study. Cupping therapy and the sham control were assigned to each participant (Figure 1). In order to reduce the carry-over effect, we implemented a counterbalanced design in this study. This design allowed half of the participants to receive cupping therapy first and then the sham control, and the other half received the sham control first and then cupping therapy. Each intervention (cupping therapy and sham control) included 2 visits with 24 h intervals for assessing the immediate (visits 2 and 4) and delayed effects (visits 3 and 5) (Figure 1). The first visit was used to determine the eligibility for this study and 75% of 10 repetition maximum (RM) biceps brachii curl of the non-dominant arm for each participant. The purpose of choosing the non-dominant arm was to minimize the effect of muscle fatigue caused by activities of daily life of a participant. There was a 1-week interval between the 2 interventions for eliminating the effect of fatigue on the subsequent intervention.

### Procedures

During the 1st visit, the participant’s weight, height, SBP, and DBP were measured. Then, the participant completed the Edinburg handedness inventory (Veale, 2014) for determination of the non-dominant arm. The participant performed biceps brachii curls without any load for 5 min. After a 3-min rest, the participant performed a series of biceps curls with different weights of dumbbells for the determination of the weight of 10RM while sitting on a bench (Hwang et al., 2016). Participants were asked “How many more repetitions do you think you can do with this weight?” If the participant could complete more than 10 repetitions, the weight would be increased until the determination of the amount of 10RM for each individual. There was a 3-min rest between each 10-repetition trial. Approximately 5 trials were performed by each participant to determine their 10RM. After that, the 75% of 10RM of biceps curls was calculated and used for the fatigue protocols (Gonzalez-Izal et al., 2010; Soares et al., 2016).

During the second visit, the participant sat on a bench and performed a 5-min warm-up followed by isolated biceps curls at 75% of 10RM until exhaustion. A metronome computer application was used to guide each participant while performing biceps curls at the pace of 15 repetitions per min. Then, one of 2 interventions (cupping therapy or sham control) was assigned according to a pre-determined counter-balanced order (Figure 1). After the biceps curls trial, the cup was placed on the biceps with negative pressure at 300 mmHg for 5 min for the experimental group. The location of the cupping cup on the biceps was the same as the location of EMG electrodes (see below). Then, the participant performed another fatigue protocol with the same number of the biceps curls as the first in order to assess the immediate effect of cupping therapy on reducing muscle fatigue while the EMG electrodes were taped to the biceps. The same number of biceps curls used in the immediate effect studies and in the delayed effect studies minimized the influences of other fatiguing factors on EMG signals. Three-lead myoelectric amplifiers (model EMG100C, Biopac Systems, Inc., Goleta, CA) were used to sample myoelectric data via bipolar surface electrodes (EL507, Biopac System, Inc., Goleta, CA). The sampling rate was set to 1,000 Hz with a 60 Hz notch option to mitigate power line interference. The EMG electrodes were placed on the line between the medial acromion and the fossa cubit at 1/3 of the way from the fossa cubit (Hermens et al., 2000).

After 24 h (third visit), the participant performed a 5-min warm-up and then the biceps curls at 75% of 10RM until exhaustion for assessing the delayed effect of cupping therapy on reducing muscle fatigue. After 1 week, the procedures conducted in the 2nd and 3rd visits were repeated for the other intervention (sham control or cupping therapy) during the 4th and 5th visits (Figure 1).

### EMG Analysis

The most commonly used algorithm to quantify muscle fatigue from EMG signals are the mean frequency (MNF) (Park and Meek, 1993), median frequency (MDF) (De Luca, 1984; Kahl and Hofmann, 2016), and spectral moments ratio (SMR) (Dimitrov et al., 2006), which usually show a monotonous trend with the increase of muscle fatigue. Mathematical modeling has demonstrated that the MNF, MDF, and SMR have good sensitivity for measuring neuromuscular fatigue (Mesin et al., 2009).

The MNF and MDF of the power spectrum are defined as

(1) $$MNF=\int_{0}^{f_{s}/2}f\cdot P\left(f\right)df/\int_{0}^{f_{s}/2}P\left(f\right)df$$

(2) $$\int_{0}^{MDF}P\left(f\right)df=\int_{MDF}^{f_{s}/2}P\left(f\right)df=\frac{1}{2}\int_{0}^{f_{s}}P\left(f\right)df$$

where *f* is frequency, *P*(*f*) the power at frequency *f*, and *f*_*s*_ the sampling frequency. During muscle fatigue, MNF and MDF decrease.

The SMR (Dimitrov et al., 2006) is defined as

(3) $$SMR=\ln\frac{M_{-1}}{M_{k}},M_{k}=\int_{f_{\min}}^{f_{\max}}f^{k}P\left(f\right)df$$

where *M* is the spectral moment of order k, *f* is frequency, *P*(*f*) the power at frequency *f*, and the parameters *k=5*, *f*_*min*_ = 5 Hz, and *f*_*max*_ = 500 were used as bounds in the calculation of SMR (Kahl and Hofmann, 2016). To investigate the progression of muscle fatigue during exercise, the short-time Fourier transform was applied to the EMG signal in segment-wise fashion. Each segment corresponded to a bout of exercise, including 10 repetitions of biceps curls. The epoch length typically used in spectral analysis of EMG signals for detecting muscle fatigue ranges between 0.25 and 2 s; thus, the parameters *k=5*, *f*_*min*_ = 5 Hz, and *f*_*max*_ = 500 were used in the calculation of SMR (Kahl and Hofmann, 2016). During muscle fatigue, SMR increases.

**FIGURE 2:**
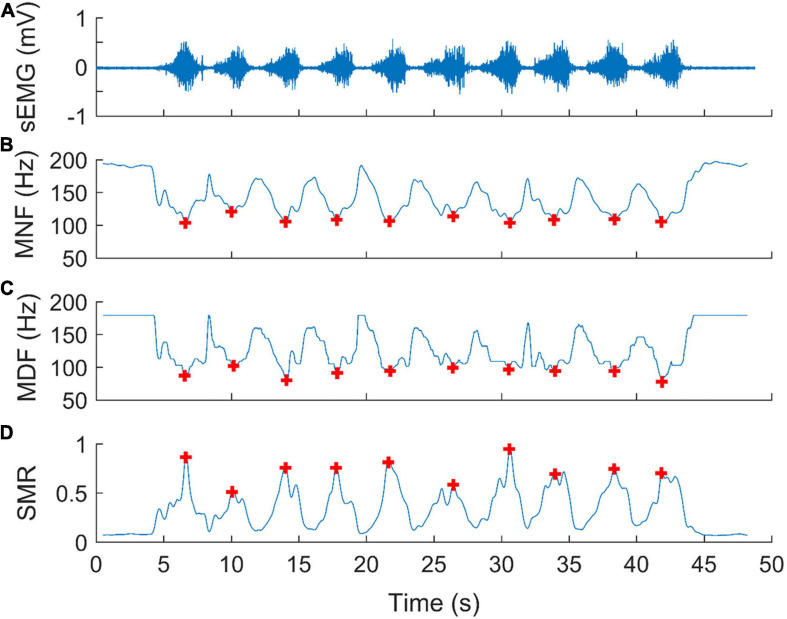
An example of spectral analysis of surface electromyographic signals (sEMG) for assessing muscle fatigue during biceps curls. **(A)** A segment of an EMG signal corresponding to a bout of exercise, including 10 repetitions of biceps curls. **(B)** Mean frequency (MNF), **(C)** median Frequency (MDF), and **(D)** spectral moments ratio (SMR) of the power spectra calculated using a Hamming window of 0.512 s (512 samples) moving in a step of 0.032 s (32 samples). Each red plus represents a global minimum of MNFs, MDFs, or the global maximum of SMRs during biceps curls at 75% of 10RM.

We computed the power spectrum of the EMG signal using a Hamming window of 0.512 s moving in a step of 0.032 s. This means that each 0.512-s epoch of EMG signal yields a power spectrum, which in turn yields a value of MNF, MDF, and SMR, respectively. Figure 2 shows an example of spectral analysis of EMG signals. Typically, during each repetition of weight training, MNF and MDF decreased with exercise progression (Figures 2B,C), while SMR exhibited an inverse trend (Figure 2D). Based on these observations, we assumed that the global minimum of MNFs and MDFs and the global maximum of SMRs during each repetition of biceps curls could reflect the degree of muscle fatigue. Therefore, we used three parameters, MNF, MDF, and SMR to evaluate the effect of cupping therapy on reducing muscle fatigue, defined as the ratio of mean value of the global minima of MNFs and MDFs or maxima of SMRs during the last10 repetitions to that during the first10 repetitions of biceps curls.

### Statistical Analysis

The difference of EMG indices (MNF, MDF, and SMR) after cupping therapy between the first 10 repetitions and the last 10 repetitions and the difference of MNF, MDF, and SMR of the immediate and delayed effects between the two interventions (cupping therapy and sham control) were examined using the Wilcoxon signed rank tests. The level of the significance was set at *p* < 0.05. The statistical analyses were implemented by using the SPSS (Version 26, Chicago, IL).

## Results

The demographic data were (mean ± standard deviation): age, 27.5 ± 6.3 years and body mass index 22.3 ± 2.6 kg/m^2^; SBP 106.6 ± 15.4 mmHg; DBP 72.3 ± 7.6 mmHg; 75% of 10RM of the non-dominant hand 12.6 ± 3.9 lb; and all participants were right handed. The number of repetitions were 7.3 ± 2.9 sets of 10 repetitions for the immediate effect studies and 4.6 ± 1.4 sets of 10 repetitions for the delayed effect studies.

**FIGURE 3:**
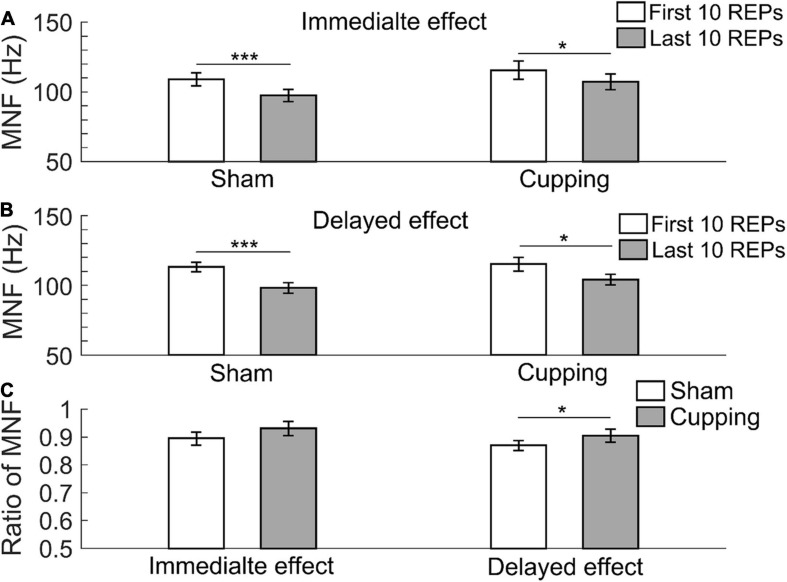
The mean frequency (MNF) of the first 10 repetitions and the last 10 repetitions of **(A)** the immediate effect and **(B)** the delayed effect of cupping therapy and sham control. **(C)** The ratios of MNF of the immediate and delayed effects after cupping therapy and sham control. The symbols * and *** indicate *p* < 0.05 and *p* < 0.001, respectively.

The MNF significantly decreased from the first 10 repetitions to the last 10 repetitions during biceps curls immediately after sham control/no intervention (first 10 repetitions of biceps curls 109.09 ± 4.72 Hz vs. last 10 repetitions 97.50 ± 4.44 Hz, *p <* 0.001) and cupping therapy (first 10 repetitions 115.63 ± 6.59 Hz vs. last 10 repetitions 107.25 ± 5.74 Hz, *p* = 0.0391 *<* 0.05) (Figure 3A); and 24 h after the sham control (first 10 repetitions 113.32 ± 3.41 Hz vs. last 10 repetitions 98.13 ± 3.85 Hz, *p <* 0.001) and cupping therapy (first 10 repetitions 115.37 ± 4.94 Hz vs. last 10 repetitions 104.12 ± 3.96 Hz, *p* = 0.0313 *<* 0.05) (Figure 3B). When comparing the change of MNF after cupping therapy to sham control, there was no immediate effect on reducing muscle fatigue (sham 0.89 ± 0.02 vs. cupping 0.93 ± 0.03, *p* = 0.0781 > 0.05). However, there was a significant delayed effect on reducing muscle fatigue (sham 0.87 ± 0.02 vs. cupping 0.91 ± 0.02, *p* = 0.0313 *<* 0.05) (Figure 3C).

**FIGURE 4:**
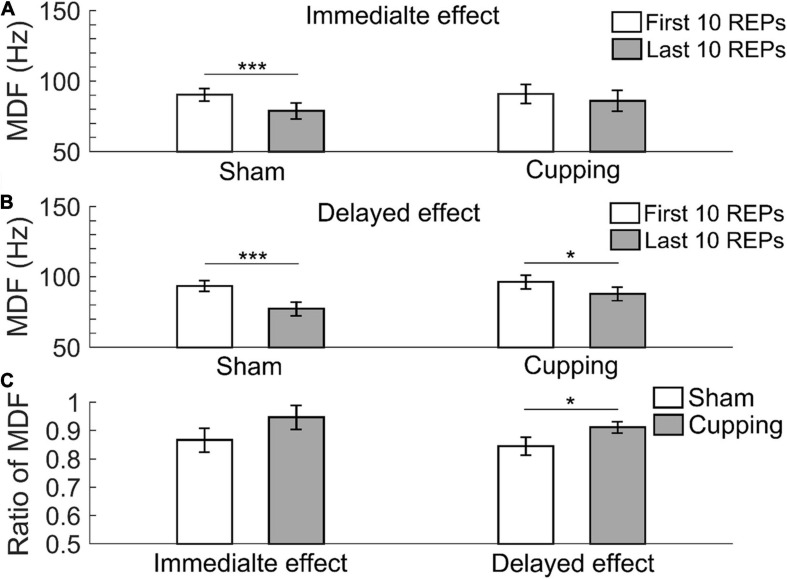
The median frequency (MDF) of the first 10 repetitions and the last 10 repetitions of **(A)** the immediate effect and **(B)** the delayed effect of cupping therapy and sham control. **(C)** The ratios of MDF of the immediate and delayed effects of cupping therapy and sham control. The symbols * and *** indicate *p* < 0.05 and *p* < 0.001, respectively.

The MDF significantly decreased from the first 10 repetitions to the last 10 repetitions immediately after the sham control (first 10 repetitions 90.37 ± 4.36 Hz vs. last 10 repetitions 78.87 ± 5.62 Hz, *p <* 0.001), but not after cupping therapy (first 10 repetitions 90.91 ± 6.59 Hz vs. last 10 repetitions 86.04 ± 7.25 Hz, *p* = 0.25 > 0.05) (Figure 4A); and 24 h after the sham control (first 10 repetitions 93.43 ± 3.82 Hz vs. last 10 repetitions 77.48 ± 5.03 Hz, *p <* 0.001) and cupping therapy (first 10 repetitions 96.32 ± 4.92 Hz vs. last 10 repetitions 87.79 ± 4.85 Hz, *p* = 0.0156 *<* 0.05) (Figure 4B). When comparing the change of MDF after cupping therapy to sham control, there was no immediate effect on reducing muscle fatigue (sham 0.87 ± 0.04 vs. cupping 0.95 ± 0.04, *p* = 0.0781 > 0.05). However, there was a significant delayed effect on reducing muscle fatigue (sham 0.85 ± 0.03 vs. cupping 0.91 ± 0.02, *p* = 0.0313 *<* 0.05) (Figure 4C).

**FIGURE 5:**
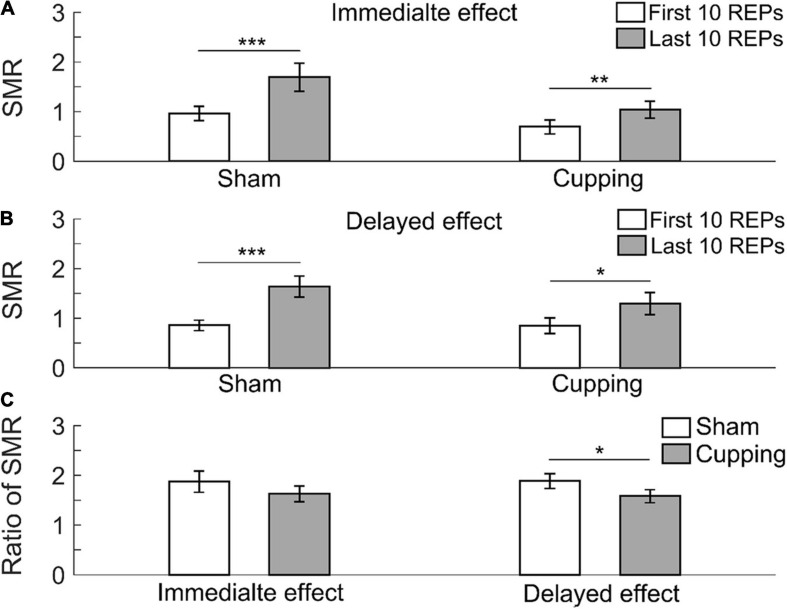
The spectral moments ratio (SMR) of the first 10 repetitions and the last 10 repetitions of **(A)** the immediate effect and **(B)** the delayed effect of cupping therapy and sham control. **(C)** The ratios of SMR of the immediate and delayed effects after cupping therapy and sham control. The symbols *, **, and *** indicate *p* < 0.05, *p* < 0.01, and *p* < 0.001, respectively.

The SMR significantly increased from the first 10 repetitions to the last 10 repetitions immediately after the sham control (first 10 repetitions 0.97 ± 0.14 vs. last 10 repetitions 1.70 ± 0.28, *p <* 0.001) and cupping therapy (first 10 repetitions 0.70 ± 0.14 vs. last 10 repetitions 1.04 ± 0.17, *p <* 0.01) (Figure 5A); and 24 h after the sham control (first 10 repetitions 0.86 ± 0.11 vs. last 10 repetitions 1.64 ± 0.22, *p <* 0.001) and cupping therapy (first 10 repetitions 0.85 ± 0.16 vs. last 10 repetitions 1.29 ± 0.22, *p* = 0.0156 *<* 0.05) (Figure 5B). When comparing the change of SMR after cupping therapy to sham control, there was no immediate effect on reducing muscle fatigue (sham 1.88 ± 0.21 vs. cupping 1.63 ± 0.16, *p* = 0.3828 > 0.05), and there was a significant delayed effect on reducing muscle fatigue (sham 1.89 ± 0.15 vs. cupping 1.58 ± 0.13, *p* = 0.0313 *<* 0.05) (Figure 5C).

## Discussion

This study demonstrated that cupping therapy had a significant delayed effect, but not an immediate effect, on reducing muscle fatigue. The use of sham control (no negative pressure) and quantitative EMG data in this study provides a better study design than previous clinical reporting using subjective outcome measures. To the best of our knowledge, this is the first study investigating the effect of cupping therapy on reducing muscle fatigue using a quantitative outcome (EMG).

In this study, a significant delayed effect, but not an immediate effect, of cupping therapy on reducing muscle fatigue may be related to the differences in removal speed of intramuscular hydrogen ions (H^+^) after cupping therapy (Sahlin et al., 1998). Cupping therapy can increase blood flow (Hou et al., 2020), which may increase clearance of H^+^. The high [H^+^] within the muscle produced by biceps curls could restrain the sarcoplasmic reticulum from releasing Ca^2+^ into sarcoplasm, and inhibit the binding of Ca^2+^ to troponin through the competitive binding of H^+^ to Ca^2+^-specific sites on troponin C (Sahlin, 1992; Fitts, 1994) and it could reduce myofibrillar ATPase activity (Metzger and Moss, 1987). These disturbances caused by H^+^ could directly affect excitation-contraction coupling and reduce cross bridge formation (MacIntosh and Rassier, 2002; Allen et al., 2008). Negative pressure of cupping therapy can elicit vasodilation and petechiae (Tham et al., 2006), which makes intramuscular H^+^ easily flow into the blood vessels of the biceps. However, the full return of muscle pH to pre-exercise levels following intensive exercise-induced fatigue may take up to 60 min or longer (Neric et al., 2009). In the present study, 5-min cupping therapy may not have been adequate to remove H^+^ completely; thus, the immediate effect after cupping therapy was not significant. As shown in Figures 3C, 4C, 5C, there were no differences in the immediate effects between the sham control and cupping therapy. Nevertheless, for the delayed effect, the ratios of MNF and MDF after cupping therapy were significantly higher than those after the sham control, and the ratio of SMR after cupping therapy was significantly lower than that of the sham control. This is because 1 day after cupping therapy may provide sufficient time for reducing [H^+^].

In addition to changes of blood flow caused by cupping therapy, the delayed effect of cupping therapy may be related to a delayed inflammatory response after cupping therapy. In the present study, the biceps curls included concentric and eccentric contractions. Muscle contraction, especially an eccentric contraction, can produce high mechanical stress on the myofibrils and damage muscle tissue, which can trigger inflammation (Goodall and Howatson, 2008; Vaile et al., 2008; Torres et al., 2012). Cupping therapy applied immediately after intensive exercise could increase local blood flow, causes the capillary to rupture, develop the ecchymosis, and induce an inflammatory response (Lowe, 2017; Al-Bedah et al., 2019). The blister and ecchymosis caused by cupping therapy may be caused by an inflammatory response (Turk and Allen, 1983). Neutrophils are the first inflammatory factor to arrive at the local tissue within 3–6 h, and then, the inducible enzyme heme oxygenase-1 (HO-1) appears after 6 h (Langlois, 2007) with the peak level in 1–3 days (Langlois et al., 2015). As a stress protein with a potent anti-inflammatory, anti-apoptotic and immunomodulatory effect, HO-1 may modulate interleukin-10 (IL-10), an anti-inflammatory cytokine (Lee and Chau, 2002; Piantadosi et al., 2011), and decrease pro-inflammatory cytokines like tumor necrosis factor-alpha (TNF-α) and interleukin-6 (IL-6) (Clerigues et al., 2012; Jamal Uddin et al., 2016). Thus, this antagonized response to inflammation caused by exercise-induced fatigue could take more than 6 h post-exercise. Therefore, the inflammation caused by cupping therapy could add to the inflammation induced by intense exercise; and the cupping procedure could augment the inflammatory response. Under this circumstance, more anti-inflammatory factors would be released into muscle tissues to modulate inflammatory reactions (Takamiya et al., 2005). This may partly explain why the delayed effect was significant but not the immediate effect. Future studies may need to assess these biomarkers to prove our speculations.

Although cupping therapy has been gaining popularity in competitive sports in recent years (Lowe, 2017), there is little scientific evidence of the effect of cupping therapy on reducing muscle fatigue. To the best of our knowledge, this is the first study to investigate the effect of cupping therapy on reducing exercise-induced muscle fatigue. Additionally, we found that cupping therapy had a significant delayed effect while not an immediate effect on neuromuscular fatigue. Thus, in sport training and competitions, an appropriate time point and duration of cupping therapy could produce better recovery effect on muscle fatigue. Lack of standard clinical practice guidelines on cupping therapy may partly contribute to the inconsistent outcomes of cupping therapy practice.

There are several limitations in this study. First, we only tested the delayed effect at 24 h after exercise-induced muscle fatigue, but did not test at longer time points (e.g., 48 and 72 h). Although the 24-h delayed effect was better than the immediate effect of cupping therapy in this study, we did not identify the optimal time point of cupping therapy for the recovery of muscle fatigue. Second, considering the safety of exercise load, the parameter of fatigue protocol for non-trained healthy adults in our study (i.e., 75% of 10RM) was set based on the intensive load (10RM) that could cause significant changes in EMG characteristic in well-trained participants. Future studies may need to examine other fatigue protocols, such as 1RM and 5RM. Third, we chose biceps curls rather than actual sports movements to induce muscle fatigue, although biceps curls consisting of dynamic concentric and eccentric contractions is much closer to the real situation of activities of daily living than the isometric contraction during maximal voluntary isometric contraction. Last, biomarkers of muscle fatigue and inflammatory response were not measured in this study. Future studies may measure these biomarkers to better understand the underlying regulatory mechanisms.

## Conclusion

The findings of this study demonstrate that there is a time effect of cupping therapy for reducing muscle fatigue. The delayed effect (24 h) of cupping therapy performed immediately after exercise was significant while the initial effect is not significant.

## Data Availability Statement

The original contributions presented in the study are included in the article/supplementary material, further inquiries can be directed to the corresponding author/s.

## Ethics Statement

The studies involving human participants were reviewed and approved by the University of Illinois at Urbana-Champaign. The patients/participants provided their written informed consent to participate in this study.

## Author Contributions

Y-KJ: conceptualization, methodology, and project administration. XH, FL, and Y-KJ: formal analysis. XH and XW: data curation. XW, LG, and Y-KJ: writing—original draft preparation. XH, XW, LG, FL, JP, and Y-KJ: writing—review and editing. All authors have read and agreed to the published version of the manuscript.

## Conflict of Interest

The authors declare that the research was conducted in the absence of any commercial or financial relationships that could be construed as a potential conflict of interest.
